# MicroRNA Expression Profiling Reveals MiRNA Families Regulating Specific Biological Pathways in Mouse Frontal Cortex and Hippocampus

**DOI:** 10.1371/journal.pone.0021495

**Published:** 2011-06-22

**Authors:** Juuso Juhila, Tessa Sipilä, Katherine Icay, Daniel Nicorici, Pekka Ellonen, Aleksi Kallio, Eija Korpelainen, Dario Greco, Iiris Hovatta

**Affiliations:** 1 Institute of Molecular Medicine Finland (FIMM), University of Helsinki, Helsinki, Finland; 2 Research Programs Unit, Molecular Neurology, Biomedicum-Helsinki, University of Helsinki, Helsinki, Finland; 3 Department of Medical Genetics, Haartman Institute, University of Helsinki, Helsinki, Finland; 4 Department of Mental Health and Substance Abuse Services, National Institute for Health and Welfare, Helsinki, Finland; 5 CSC, IT Center for Science, Espoo, Finland; 6 Department of Bioscience and Nutrition, Karolinska Institutet, Stockholm, Sweden; Instituto Nacional de Câncer, Brazil

## Abstract

MicroRNAs (miRNAs) are small regulatory molecules that cause post-transcriptional gene silencing. Although some miRNAs are known to have region-specific expression patterns in the adult brain, the functional consequences of the region-specificity to the gene regulatory networks of the brain nuclei are not clear. Therefore, we studied miRNA expression patterns by miRNA-Seq and microarrays in two brain regions, frontal cortex (FCx) and hippocampus (HP), which have separate biological functions. We identified 354 miRNAs from FCx and 408 from HP using miRNA-Seq, and 245 from FCx and 238 from HP with microarrays. Several miRNA families and clusters were differentially expressed between FCx and HP, including the miR-8 family, miR-182|miR-96|miR-183 cluster, and miR-212|miR-312 cluster overexpressed in FCx and miR-34 family overexpressed in HP. To visualize the clusters, we developed support for viewing genomic alignments of miRNA-Seq reads in the Chipster genome browser. We carried out pathway analysis of the predicted target genes of differentially expressed miRNA families and clusters to assess their putative biological functions. Interestingly, several miRNAs from the same family/cluster were predicted to regulate specific biological pathways. We have developed a miRNA-Seq approach with a bioinformatic analysis workflow that is suitable for studying miRNA expression patterns from specific brain nuclei. FCx and HP were shown to have distinct miRNA expression patterns which were reflected in the predicted gene regulatory pathways. This methodology can be applied for the identification of brain region-specific and phenotype-specific miRNA-mRNA-regulatory networks from the adult and developing rodent brain.

## Introduction

MicroRNAs (miRNAs) are highly conserved small regulatory molecules that cause post-transcriptional gene silencing by base pairing with target messenger RNA (mRNA) [1]. MiRNAs are processed from primary transcripts into ∼70-nt stem-loop precursors in the nucleus by Drosha-DGCR (DiGeorge Critical Region) complex, and then further cleaved by the Dicer enzyme in the cytoplasm into ∼23-nt functional sequences [2]. Mature mammalian miRNAs bind to their specific target sequences on the 3′-untranslated region (UTR) of mRNAs by imperfect base pairing, leading to mRNA cleavage or translational repression [3], [4]. Because one miRNA may have hundreds of target mRNAs, each miRNA has wide-reaching effects on gene expression, and together with their target mRNAs they form non-linear gene regulatory networks.

MiRNAs have diverse functions in the brain, including regulation of neuronal development and differentiation, synapse formation precursorand plasticity [5], [6], [7]. MiRNA expression levels seem to be dynamic in the mammalian brain since they are altered by environmental stimuli. For example, acute stress increases let-7a, miR-9 and miR-26a/b expression levels in the mouse frontal cortex (FCx), but not in the hippocampus (HP) [8], and miR-212 signalling in the rat striatum has a role in determining vulnerability to cocaine addiction [9]. Therefore, it is not surprising that miRNAs have been implicated in the etiology of several brain disorders, including Alzheimer's disease [10], [11], Parkinson's disease [12], schizophrenia [13], aggressive behavior [14], depression [15], and anxiety disorders [16], [17].

MiRNAs play an important role during development, and some miRNAs have specific temporal and spatial expression patterns (e.g. [18], [19], [20]), suggesting that they control particular developmental steps. In the adult brain, many miRNAs are widely expressed, but there are also miRNAs that show enrichment in specific brain regions [21], [22]. There is evidence that region-specific expression patterns of some miRNAs coincide with regional expression of their predicted target mRNAs [20], although it is currently not known how widely this phenomenon applies. However, the region-specificity of mRNA expression is well-characterized in the mouse brain [23], [24], [25], and since miRNAs are estimated to regulate the expression level of about 30% of the mammalian mRNAs [26], it is likely that at least some regulatory networks involving mRNAs and non-coding regulatory RNAs have region-specific expression patterns.

Brain region-specific miRNA expression patterns of the adult mouse have earlier been investigated with in situ hybridization, qPCR, and microarrays [20], [21]. The recent development of massively parallel sequencing techniques has enabled profiling of all expressed miRNAs in a given brain nucleus with a wide quantitative range, which is especially important in the brain where many genes are expressed on a relatively low level [23]. Therefore, we set out to investigate miRNA expression patterns in two brain regions, FCx which is involved in the processing of cognitive information and HP responsible for the context specificity and learning and memory. MiRNA expression patterns from the adult mouse brain were determined by miRNA-Seq using the Illumina Genome Analyzer and by Affymetrix miRNA microarray. Interestingly, several miRNA families and clusters comprising of multiple miRNAs were differentially expressed between FCx and HP. To identify biological pathways regulated by differentially expressed miRNAs, we predicted their target genes and carried out pathway analysis. Different pathways were identified as being enriched in FCx and HP emphasizing molecular diversity of these brain regions.

## Results

### Identification of miRNAs from FCx and HP by miRNA-Seq

To determine miRNA expression patterns in FCx and HP, we carried out miRNA-Seq using the Illumina Genome Analyzer II. We developed an indexing protocol that is based on the 5′ adaptor modification. Three cDNA libraries prepared from the same totalRNA and carrying different 6 bp indexes were sequenced individually, totaling three FCx and three HP samples (Figure S1). To test how the indexes performed when used together, we sequenced one library from both brain regions that contained equal amounts of each three separate indexes pooled together. Obtained sequences were trimmed and mapped against the miRBase, log2-transformed and normalized to obtain a relative miRNA expression level for all previously known miRNAs (see materials and methods for details).

It is known that indexes with different nucleotide sequences might ligate to miRNAs with different efficacy [27]. To investigate if there was a bias in miRNA expression patterns due to the three different indexes, we calculated correlation coefficients for the pairwise comparisons of the three indexes (Figure S2). The correlation coefficients varied from R^2^ = 0.803 to 0.907 indicating no major ligation bias. Also, correlation between individually run and pooled samples was high in both FCx (R^2^ = 0.976) and HP (R^2^ = 0.978). Because indexing did not significantly alter miRNA expression patterns, differently indexed libraries were treated as technical replicates in the subsequent analyses.

Sequencing of the libraries resulted in on average 6.22 million (FCx) and 5.65 million (HP) short reads per library (Table 1), which corresponded to 354 detected previously known miRNAs in FCx and 408 in HP.

**Table 1:** Bioinformatic workflow of miRNA-Seq data and the number of obtained reads.

	Frontal Cortex	Hippocampus
**# of technical replicates**	6	6
**# of pre-filtered reads after base calling**	6 223 794	5 652 753
**# of sorted reads with indexes**	5 554 153	5 246 449
**Indexing efficiency (%)**	89.2	92.8
**Distribution of indexes (%):Index 3 (UUAGGC)**	40.5	23.5
** Index 7 (CAGAUC)**	33.7	36.8
** Index 11 (GGCUAC)**	25.8	39.7
**# of trimmed reads without mitochondrial and ribosomal sequences**	1 778 620	4 387 680
**# of reads aligning against mouse genome**	1 043 842	3 546 816
**# of reads aligning against miRBase**	626 398	3 053 964
**# of known miRNAs**	415	446
**# of reads aligning against miRBase with ≥2 reads**	626 329	3 053 908
**# of known miRNAs with ≥2 reads**	354	408

### Identification of miRNAs from FCx and HP by Affymetrix miRNA array

To compare miRNA expression patterns obtained by miRNA-Seq with an independent method, we analyzed FCx and HP miRNA expression also with Affymetrix GeneChip® miRNA arrays. They contain four probe sets for each of the 609 mouse miRNAs from the miRBase v11. Three biological replicates from FCx and HP were analyzed on independent arrays. We detected 245 miRNAs from FCx and 238 from HP.

### Comparison of the miRNA expression patterns obtained by miRNA-Seq and microarrays

**Figure 1:**
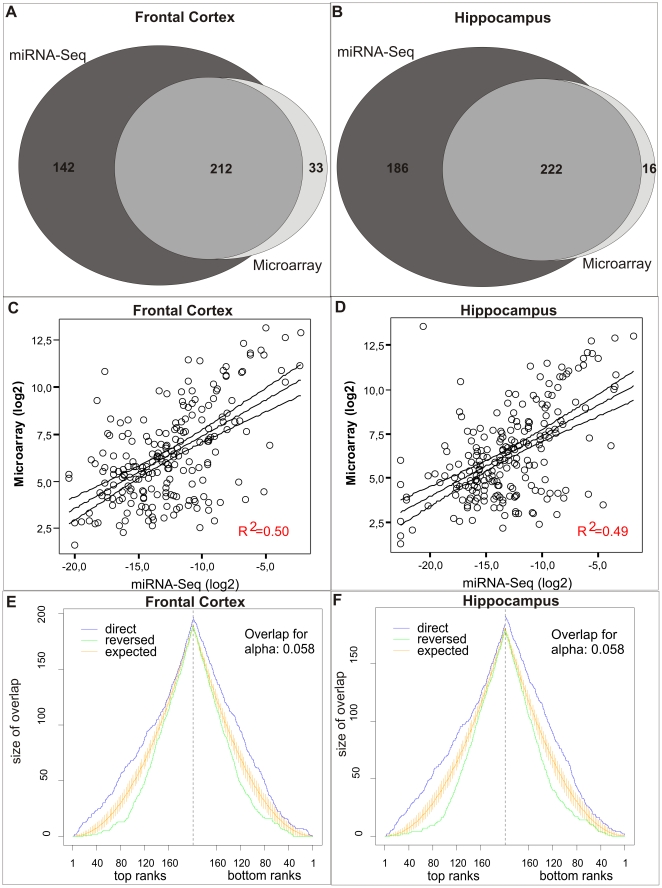
Number of miRNAs detected by miRNA-Seq and microarrays, and comparison of the two platforms. Number of miRNAs detected by miRNA-Seq (**a**) in frontal cortex and (**b**) in hippocampus. (**c and d**) Comparison of normalized count numbers from miRNA-Seq with normalized signal intensity from microarrays in frontal cortex and hippocampus samples for miRNAs detected with both platforms. R^2^ = Spearman correlation coefficient for pairwise comparison. Similarity of ranked expression value lists for (**e**) frontal cortex and (**f**) hippocampus resulting from miRNA-Seq and microarrays as analyzed by the OrderedList package in R/Bioconductor. Expected refers to the expected overlap and 95% confidence interval is shown. Both lists are more similar than expected by chance in the direct order (from the largest to the smallest expression value; P<0.001).

The number of miRNAs detected by both platforms was 212 in FCx and 222 in HP (Figure 1A and B). We next analyzed the similarities of the miRNA expression patterns obtained by the two methods using two approaches. First, normalized count numbers obtained from miRNA-Seq were plotted against normalized microarray signal levels (Figure 1 C and D). The Spearman correlation coefficient between the two methods was 0.50 in FCx and 0.49 in HP. Second, we ranked the miRNAs from both platforms according to their normalized expression levels and compared the similarities between the ordered lists giving more weight on the miRNAs that were expressed on a high level (top ranks) or low level (bottom ranks). According to the rank test, miRNA expression patterns from miRNA-Seq and microarrays were significantly similar to each other both in FCx and HP (p-value <0.001) (Figure 1 E and F). These analyses suggested good correlation between the two methods, as shown before [28].

**Figure 2:**
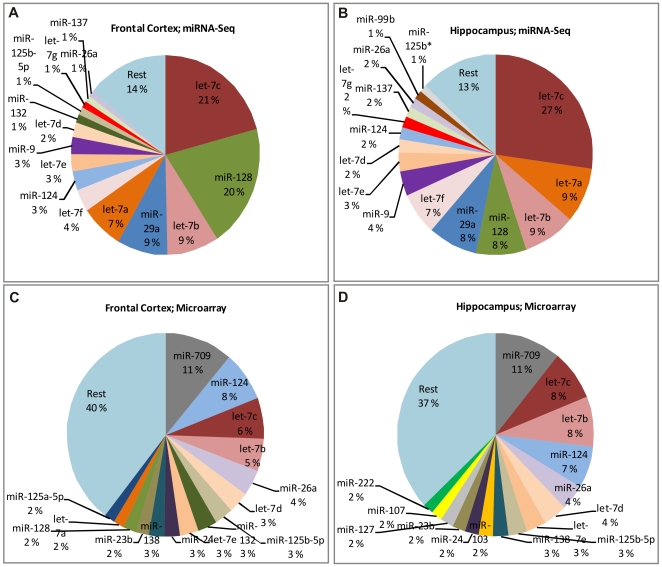
The most abundant miRNAs in frontal cortex and hippocampus. Distribution of normalized counts (miRNA-Seq) or signal intensity (microarrays) for the 15 most abundant miRNAs. (**a**)> miRNA-Seq from frontal cortex, (**b**) miRNA-Seq from hippocampus, (**c**) microarray from frontal cortex, and (**d**) microarray from hippocampus.

Next, we examined the relative distributions of the most abundant miRNAs by platform. Of the 15 most abundant miRNAs 10 were shared between both methods in FCx and seven in HP (Figure 2). In miRNA-Seq data, the 15 most abundant miRNAs constituted 86% of all counts in FCx and 87% in HP, while in the microarray data, the 15 most abundant miRNAs constituted only 60% of the total signal in FCx and 63% in HP. As an example, let-7c was highly expressed in both platforms but the proportion of its expression level was much higher in miRNA-Seq (21% in FCx and 27% in HP of all counts) compared to its proportional expression detected by microarrays (6% in FCx and 8% in HP of all signal). This finding most likely reflects differences between the sample preparation, including a PCR amplification step in the miRNA-Seq but not in the microarray protocol, and the fact that the microarrays contain probes for several species putatively allowing cross hybridization and dilution of the signal of the conserved miRNAs.

### Several miRNA families and clusters are differentially expressed between FCx and HP

MiRNAs that are differentially expressed between FCx and HP are expected to contribute to functional differences between the brain regions. Therefore, we identified miRNAs that are differentially expressed (p<0.05) between FCx and HP by miRNA-Seq, microarrays or both (Table S1). We detected 36 differentially expressed miRNAs (20 upregulated in FCx and 16 in HP) by miRNA-Seq and 55 (28 upregulated in FCx and 27 in HP) by microarrays. The overlap of the differentially expressed miRNAs detected in both platforms, however, was relatively low with nine miRNAs in FCx and four in HP. P-values derived from the microarray and miRNA-Seq analyses are not independent, but correlated. For that reason, widely accepted multiple testing correction methods, such as Holm-Bonferroni [29] and Benjamini-Hochberg [30], produce strongly conservative and unrealistic results. Applying Benjamini-Hochberg correction reduced the number of differentially expressed miRNAs with FDR <0.05 to four in the case of microarray and 13 in miRNA-Seq. Taking into account the nature of the data, multiple testing correction was not used as a selection criterion for this study.

Interestingly, we identified several miRNA families and clusters being differentially expressed between FCx and HP (Table 2). These included miR-8 family overexpressed in FCx and comprising of miRNAs from two chromosomal clusters, miR-429, miR-200a, miR-200a*, miR-200b, and miR-200b* from chromosome 4, and miR-141 and miR-200c from chromosome 6. Also, a chromosome 6 cluster with miR-182, miR-96, and miR-183 and a chromosome 11 cluster with miR-212 and miR-312 were expressed on a higher level in FCx. Mir-34 family transcribed from a chromosome 9 cluster and including miR-34c, miR-34c*, miR-34b-3p, and miR-34b-5p was expressed on a higher level in HP. Three miRNAs in FCx (miR-128, miR-129-5p, and miR-344) and one miRNA in HP (miR-7a) map to two chromosomal loci (Table S1). These data suggest contribution of specific miRNA clusters and families in the region-specific miRNA expression patterns. This finding is supported by previous observations regarding miRNA clusters regulating specific signaling pathways [22], [31].

**Table 2:** MiRNA families and clusters differentially expressed between frontal cortex and hippocampus.

miRNAs over- expressed in FCx	Chr	Cluster #	Detected by	Expression level in FCx by MA	Expression level in HP by MA	MiRNA-Seq FCx count concentration	MiRNA-Seq HP count concentration	Fold change	p-value
mmu-miR-429	4	1	MA; MS	3.57	2.05	7.46E-04	2.32E-05	2.86; 32.16	5.3E-03; 1.1E-08
mmu-miR-200a	4	1	MA; MS	3.45	1.56	1.36E-03	2.58E-05	3.69; 52.59	1.3E-02; 2.6E-10
mmu-miR-200a*	4	1	MS			1.04E-05	3.18E-07	32.86	4.3E-05
mmu-miR-200b	4	1	MA; MS	2.53	1.22	8.42E-04	2.62E-05	2.48; 32.19	5.0-E03; 1.1E-08
mmu-miR-200b*	4	1	MS			3.46E-05	8.20E-07	42.85	1.1E-07
mmu-miR-141	6	2	MS			4.16E-05	3.63E-07	118.58	8.2E-09
mmu-miR-200c	6	2	MA; MS	4.19	0.95	1.41E-04	5.44E-07	9.43; 263.86	5.6E-03; 7.8E-13
mmu-miR-182	6	3	MA; MS	5.47	1.81	5.38E-05	5.09E-07	12.58; 103.93	3.5E-04; 1.3E-09
mmu-miR-96	6	3	MS			8.74E-06	8.78E-16	FCx only	1.8E-05
mmu-miR-183	6	3	MS			5.10E-05	1.47E-07	336.12	4.9E-11
mmu-miR-212	11	4	MA; MS	8.91	8.28	4.54E-04	1.40E-04	1.55; 3.24	1.3E-02; 2.6E-02
mmu-miR-132	11	4	MA; MS	11.71	10.71	1.34E-02	3.56E-03	2.00; 3.77	1.8E-03; 1.2E-02
**miRNAs overexpressed in HP**	**Chr**	**Cluster #**	**Detected by**	**Expression level in FCx by MA**	**Expression level in HP by MA**	**MiRNA-Seq FCx count concentration**	**MiRNA-Seq HP count concentration**	**Fold change**	**p-value**
mmu-miR-34c	9	5	MA; MS	4.12	7.20	4.05E-05	1.42E-04	8.44; 3.49	1.3E-03; 2.0E-02
mmu-miR-34c*	9	5	MA	3.30	6.00			6.50	4.1E-04
mmu-miR-34b-3p	9	5	MA; MS	2.76	4.87	2.30E-06	1.49E-05	4.31; 6.59	7.3E-03; 5.6E-03
mmu-miR-34b-5p	9	5	MS			1.55E-06	1.61E-05	9.77	3.1E-04

Differentially expressed (P<0.05) miRNAs between frontal cortex and hippocampus and belonging to miRNA families or clusters are shown. Microarray data is given as average normalized (log transformed) signal intensity. MiRNA-Seq count concentration refers to normalized expression levels from edgeR. Please see Table S1 for a full list of differentially expressed miRNAs. FCx  =  frontal cortex, Chr  =  chromosome, Start  =  start position of the sequence in genomic alignment, End  =  end position of the sequence in genomic alignment, AS  =  antisense, S  =  sense, MS  =  miRNA-Seq, MA  =  microarray.

### Visualization of miRNA-Seq reads in their genomic context

**Figure 3:**
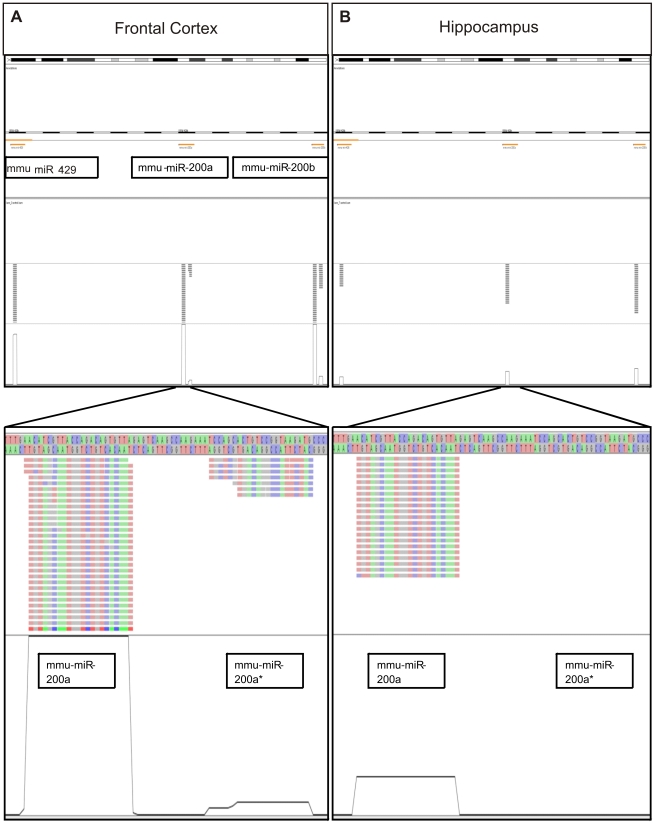
Visualization of miRNA-Seq reads aligning to the chromosome 4 cluster of miR-8 family. The Chipster tool was used to visualize the miR-8 family members located on chromosome 4 (miR-429, miR-200a, miR-200a*, miR-200b and miR200b*). As an example one sample from (**a**) frontal cortex and one sample from (**b**) hippocampus were chosen (both sequenced with Illumina index # 11). The upper half shows the number of sequences detected in the genomic region on a broad scale. The lower half zooms in on the reads detected for miRNA-200a and miR-200a*. The different colors represent different nucleotides, making single nucleotide alterations (e.g., isomirs, single nucleotide polymorphisms, or sequencing errors) easier to detect. Finally, the line graph represents the sequence coverage for each brain region before normalization.

To inspect the miRNA clusters in their genomic context we added support for genomic alignments of miRNA-Seq reads in the open source software Chipster Viewer (http://chipster.csc.fi/beta/). The interactive genome browser of Chipster Viewer allows the visualization of next generation sequencing data such as ChIP-Seq, RNA-Seq and exome-Seq in the genomic context using Ensembl annotations. It also contains preprocessing functionality which converts SAM files to BAM format and performs sorting and indexing for BAM files. As an example, Figure 3 shows visualization of miR-8 family (miR-429, miR-200a, miR-200a*, miR-200b and miR200b*) located on chromosome 4.

### Target prediction of differentially expressed miRNAs

**Figure 4:**
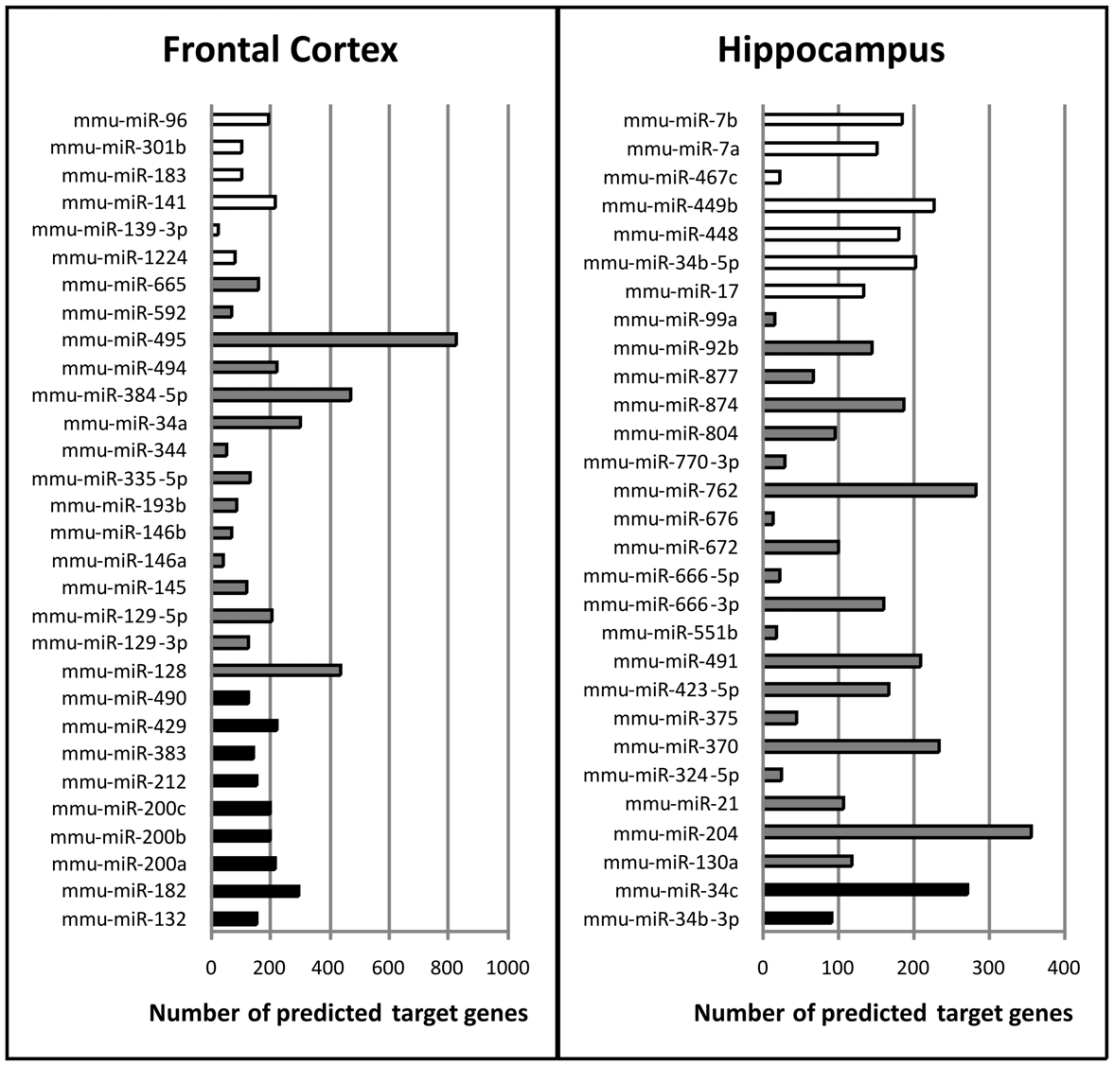
Number of predicted target genes for each miRNA differentially expressed between frontal cortex and hippocampus. All miRNAs with differential expression (P<0.05) between frontal cortex and hippocampus are shown. Left panel lists miRNAs overexpressed in frontal cortex and right panel miRNAs overexpressed in hippocampus. White bars denote miRNAs detected with miRNA-Seq, gray bars miRNAs detected by microarrays, and black bars miRNAs detected by both platforms.

To identify target mRNAs for the differentially expressed miRNAs, we carried out target prediction for all miRNAs detected by either microarray or miRNA-Seq with differential expression between FCx and HP. Out of the 39 miRNAs expressed on a higher level in the FCx and 39 expressed on a higher level in the HP, nine and 10, respectively, were star miRNAs (Table S1). Since miRWalk tool used for the target predictions does not consider star sequences, they were omitted from further analyses. Number of target genes predicted for each differentially expressed miRNA varied from 12 (miR-676) to 826 (miR-495), with an average of 190 for miRNAs overexpressed in FCx and 132 in HP (Figure 4). The majority of the predicted target genes were specific for the corresponding brain region, however, 24% of target genes of miRNAs detected by microarrays, 11% of those detected by miRNA-Seq, and 10% of those detected by both methods were shared by both brain regions (data not shown).

### Pathway analysis of target mRNAs reveals biological networks specific for each brain region

**Figure 5:**
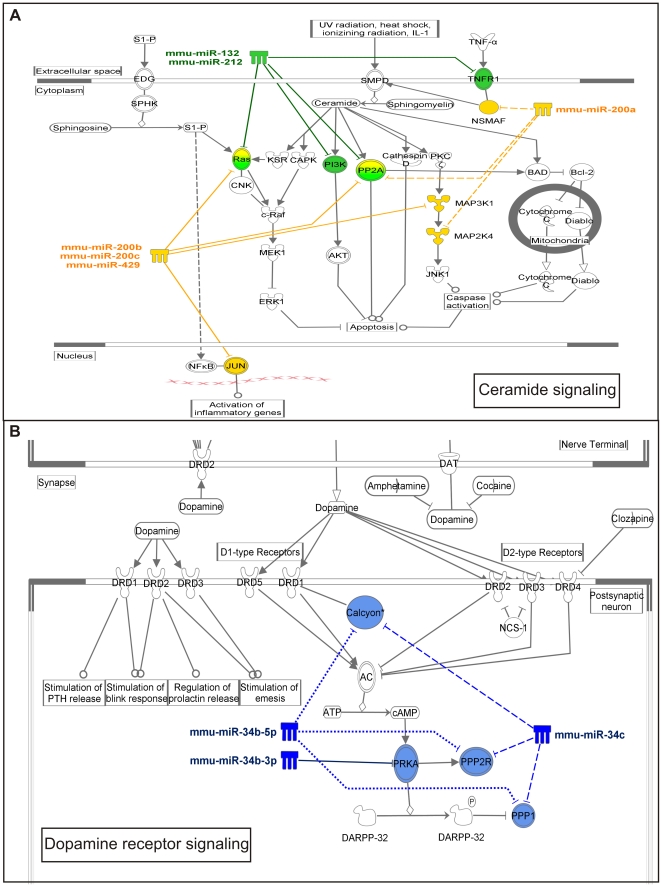
Predicted signaling pathways regulated by differentially expressed miRNA-families in frontal cortex and hippocampus. Pathway analysis of predicted target genes of differentially expressed miRNA families and clusters was carried out to assess their putative biological functions. Specific pathways that are predicted to be regulated by several miRNAs from the same family/cluster were identified (for a list of pathways see Table S2). (**a**) As an example, ceramide signaling pathway composed of miRNAs from miR-8 family and their predicted target genes is shown. (**b**) Dopamine signaling pathway including miR-34 family members and their predicted target genes.

Gene regulatory networks composed of miRNAs and their target mRNAs are expected to be important for the biological function of the specific brain regions. Therefore, using Ingenuity Pathway Analysis software, we carried out pathway analysis for miRNAs that were differentially expressed between FCx and HP and that belonged to miRNA clusters or families. These included miR-8, miR-132, and miR-34 families and the miR-182|miR-96|miR-183 cluster. The analysis was carried out simultaneously for all miRNAs belonging to the family or cluster, and the significantly over-represented pathways (P<0.05) for each of them are shown in Table S2. Interestingly, specific biological pathways were predicted to be regulated by several miRNAs from the same family/cluster. Only the ERK/MAPK signaling pathway was predicted to be regulated by miRNAs that are expressed in both FCx and HP. Figure 5 shows two example pathways, ceramide signaling regulated by two miRNA families overexpressed in FCx and dopamine signaling regulated by miRNAs overexpressed in HP.

## Discussion

Understanding the function of miRNAs in the adult brain requires investigation of brain region-specific miRNA expression patterns. MiRNA-Seq provides a new and attractive method to identify all miRNAs expressed in a given sample [32], [33], [34]. For that reason, we set up a miRNA-Seq protocol with associated bioinformatic workflow to identify miRNA expression patterns in two brain regions, FCx and HP. MiRNA-Seq produces millions of short sequences per run, a coverage which is not needed for the profiling of miRNAs. Therefore, application of indexing with short tags can increase throughput and reduce costs. At the time of this study, commercial sample prep kits with indexing were not available for the Illumina Genome Analyzer, and therefore we modified the Illumina small RNA sample prep method slightly and attached six-base-pair indexes to the 5′ adaptor sequence. This indexing strategy performed well and produced highly comparable results between identical samples sequenced three times with different indexes. We also set up a bioinformatics workflow for straightforward analysis of miRNA-Seq data. As part of this workflow and to visualize the genomic position of aligned miRNA-Seq reads, we added support for miRNA-Seq data in the open source software Chipster Viewer. It provides a user-friendly and efficient way to visualize data from different massively parallel sequencing applications such as ChIP-Seq, RNA-Seq and exome-Seq.

We compared miRNA expression patterns obtained by miRNA-Seq with those obtained by Affymetrix miRNA arrays. Correlation on the miRNA expression level was moderate (R^2^ = 0.50 in FCx and 0.49 in HP), as published earlier [28], [35]. Interestingly, there were some differences in the relative abundance of some miRNAs between the platforms. For example, miR-709 was the most abundant miRNA in FCx and HP as detected by microarrays. However, in miRNA-Seq it was not detected at all in FCx and with very low abundance (0–1 counts per library) in HP. Conversely, miR-9 was among the most expressed miRNAs in miRNA-Seq data but poorly detected by microarrays. These differences might be partly attributed to the multi-organism probe content on the Affymetrix array, as miR-709 is represented by mouse-specific probe sets, while miR-9 has similar probe sets for 23 different organisms, all of which were called “present” in our dataset. Another difference between the methods is that miRNA-Seq sample prep contains a PCR amplification step which is not present in the microarray protocol. Overall, a larger number of miRNAs were detected by miRNA-Seq compared to microarrays. Due to the differences between the platforms, in the downstream analyses we concentrated on the set of miRNAs that were detected by both methods.

Thirteen miRNAs were differentially expressed between FCx and HP and detected by both platforms. Nine were upregulated in FCx and four in HP. Interestingly, most of these differentially expressed miRNAs belonged to miRNA families, including miR-8 and miR-132 families overexpressed in FCx and miR-34 family overexpressed in HP, or miRNA clusters transcribed from the same locus (miR-182|miR-183|miR-96 cluster overexpressed in FCx). It has been established that a large proportion of mammalian miRNAs cluster on chromosomes [36], [37], and that miRNAs from the same cluster tend to be co-transcribed implying polycistronic transcription [38]. Interestingly, it seems that a miRNA cluster may regulate functionally related genes within a biological pathway. For example, miR17|miR-92 cluster is overexpressed in *Myc*-induced tumor and regulates two genes involved in angiogenesis, *TSP1* and *CTGF* [39]. The miR-182|miR-183|miR-96 cluster, also identified in our study, has previously been shown to regulate insulin signaling pathway, with miR-96 and miR-183 mainly regulating *Irs1* and miR-182 targeting *Rasa1* and *Grb2* [31]. Our data also support this notion. For example, in the ceramide pathway (Figure 5), miR132 and miR-212 were predicted to regulate *Ras, Pi3k, Pp2a*, and *Tnfr1*, miR-200a to regulate *Nsmaf, Pp2a*, and *Map2k4*, and miR-200b, miR-200c, and miR-429 to regulate *Ras, Pp2a, Map3k1*, and *Jun*. In the dopamine pathway (Figure 5) miR-34b-5p was predicted to regulate Calcyon, Ppp2r, and Ppp1, miR-34b-3p to regulate Prka, and miR-34 c to regulate *Calcyon*, *Ppp2r* and *Ppp1*.

We identified the ceramide signaling pathway by investigating the predicted biological target pathways of miR-8 and miR-132 families, both of which were expressed on a higher level in FCx compared to HP. Therefore, it is assumed that this pathway is repressed in FCx. Sphingolipids, including ceramide, play an important role in signal transduction in the membranes of neurons, either by modulating the localization and activation of membrane-associated receptors, or by acting as precursors of bioactive lipid mediators [40]. Ceramides are activated by inflammatory cytokines or oxidative stress, and participate in various physiological processes including cell proliferation, cell differentiation, and apoptosis [40]. Excessive production of ceramides, especially in HP, have been implicated in the pathological death of neurons that occurs in ischemic stroke [41], Parkinson's disease [42], and Alzheimer's disease [40].

Another interesting pathway we identified is the dopamine receptor 1/calcyon signaling pathway regulated by members of the miR-34 family expressed on a higher level in HP compared to FCx. Calcyon is a single transmembrane protein that regulates dopamine receptor 1 signaling. Genetic variants within the Calcyon gene (*DRD1IP*) associate with attention deficit hyperactivity disorder (ADHD) [43], and therefore it is interesting that transgenic mice overexpressing human calcyon cDNA show locomotor hyperactivity and reduced anxiety-like behavior [44]. In the postmortem brains of schizophrenia patients, calcyon is up-regulated in the cortex and thalamus [45], [46]. Therefore, this pathway clearly plays an important role in the etiology of neuropsychiatric phenotypes.

In conclusion, miRNA-Seq proved to be a well suited method for the analysis of miRNA expression patterns of specific brain nuclei. As part of this study, we make publically available both miRNA-Seq and microarray datasets of adult mouse FCx and HP which are expected to benefit a number of neuroscientists and bioinformaticians. Several miRNA families and clusters were shown to be differentially expressed between FCx and HP. Biological pathways these families or clusters are predicted to target are highly specialized. Furthermore, we provide further support for the notion that members of the same miRNA family or cluster target several mRNAs within a biological pathway. Our work provides a starting point for a systematic analysis of brain region-specific miRNA regulatory networks in the adult brain to better understand their involvement in various cellular functions and disease phenotypes.

## Materials and Methods

### Animals and tissue collection

All animal care conformed to the European Communities Council Directive 86/609/EEC. According to the Finnish legislation (act on the use of animals for experimental purposes 62/2006) tissue collection as a terminal procedure does not require ethics committee approval, however, the University of Helsinki internal approval for animal use (KEK09–044) was obtained as required. Six adult male C57BL/6J (Charles River) mice were obtained at the age of seven weeks and singly-housed for one week before dissections. Food and water was available ad libitum, and mice were kept on a 12-h light-dark schedule (light period 6 am-6 pm). After one week acclimatization mice were sacrificed by cervical dislocation at 9 am. FCx was dissected from a coronal slice spanning Bregma −3.5– −1.5. HP was dissected as described earlier [47]. Tissues were snap frozen in liquid nitrogen and stored at −80°C until RNA isolation.

### Total RNA extraction

Tissue was homogenized using lysing matrixD tubes and the Fastprep instrument (Q.Biogene, Carlsbad, CA) according to the manufacturer's recommendation. The extraction of total RNA was performed using the TriReagent (Molecular Research Center, Cincinnati, OH) also according to the manufacturer's instructions. RNA pellets were eluted to sequencing grade water (Amresco, Solon, OH), and the quality of total RNA analyzed by the Bioanalyzer Nano 6000 chip (Agilent Technologies, Santa Clara, CA). Only samples with an RNA Integrity Number (RIN) greater than 9.0 were used for further experiments.

### Small RNA library preparation

Small RNA library preparation was based on Illumina's Small RNA Sample Prep kit v1.0 (Illumina Inc, San Diego, CA), except that we added an index sequence to the 5′ adaptor as described below. All adaptor and primer sequences are based on Illumina's oligonucleotide sequences (© 2006–2008 Illumina, Inc. All rights reserved). Three Illumina indexes were selected, all of which start with the same nucleotide and differ from each other by at least two nucleotides. FCx and HP samples from three mice were pooled (Figure S1). Six samples were processed: 1) FCx with Illumina index # 3, 2) FCx with Illumina index # 7, 3) FCx with Illumina index # 11, 4) HP with Illumina index # 3, 5) HP with Illumina index # 7, and 6) HP with Illumina index # 11. For each sample, 10 µg of total RNA was size fractionated using a 15% Novex gel (Invitrogen, Carlsbad, CA) and fractions corresponding to 15–35 nucleotides were excised. RNA was eluted by shaking the gel slices in 300 µl of 0.3 M NaCl for 4 h at RT. The resulting gel slurries were passed through a Spin-X filter column (Corning, NY). 750 µl of 100% ethanol and 1 µl of mussel glycogen (20 mg/ml; Roche) were added and the samples were precipitated for 30 minutes at −80°C. Samples were centrifuged 14000 rpm for 25 minutes at 4°C. After washing with 85% ethanol, pellets were allowed to air dry at 25°C, and the samples were resuspended in 5.7 µl of sequencing grade water (Amresco, Solon, OH).

The 5′ RNA adaptors containing three different six-nucleotide tags were ordered from Metabion GmbH (Martinsried, Germany). Indexing was based on the multiplexing protocol used for DNA sequencing by Illumina (Oligonucleotide sequences © 2006–2008 Illumina, Inc. All rights reserved). The sequences of the 5′ RNA adaptors were: Index 3 (5′-GUUCAGAGUUCUACAGUCCGACGAUC *UUAGGC*-3′); Index 7 (5′-GUUCAGAGUUCUACAGUCCGACGAUC *CAGAUC*-3′); and Index 11 (5′-GUUCAGAGUUCUACAGUCCGACGAUC *GGCUAC-*3′). These 5′RNA adaptors (1.3 µl; 5 µM) were ligated to the small RNA samples with T4 RNA ligase 1 (1.0 µl; New England Biolabs; NEB; Ipswich, MA) in the presence of RNase inhibitor (1.0 µl; NEB) for 6 h at 20°C. The ligation reaction was stopped by the addition of 2× RNA loading dye (Fermentas GmbHSt. Leon-Rot, Germany). The ligated RNA was size fractionated using a 15% Novex gel, and a 40–75 nucleotide fraction was excised. RNA was eluted from the excised gel slices by shaking them in 300 µl of 0.3 M NaCl for 4 h at RT. The resulting gel slurries were passed through a Spin-X filter column. 750 µl of 100% ethanol and 1 µl of mussel glycogen (20 mg/ml) were added and the samples were precipitated for 30 minutes at −80°C. The samples were immediately centrifuged at 14000 rpm for 25 minutes at 4°C. After washing with 85% ethanol, the pellets were allowed to air dry at 25°C, and the samples resuspended in 6.4 µl of sequencing grade water.

The 3′ RNA adaptor (0.6 µl; 10 µM; 5′-pUCGUAUGCCGUCUUCUGCUUGidT-3′; p, phosphate; idT, inverted deoxythymidine; Metabion) was subsequently ligated to the precipitated RNA using the same reaction conditions as in the 5′ ligation step. The ligated RNA was size fractionated using a 10% Novex gel, and the 60–100 nucleotide fraction was excised. RNA was eluted from the excised gel slices by shaking them in 300 µl of 0.3 M NaCl for 4 h at RT. The resulting gel slurries were passed through a Spin-X filter column. 750 µl of 100% ethanol and 1 µl of mussel glycogen (20 mg/ml) were added and the samples were precipitated for 30 minutes at −80°C. The samples were immediately centrifuged at 14000 rpm for 25 minutes at 4°C. After washing with 85% ethanol, the pellets were allowed to air dry at 25°C, and the samples resuspended in 4.5 µl of sequencing grade water. The products containing miRNAs flanked by 5′ and 3′ adaptors were converted to cDNA using Superscript II reverse transcriptase (Invitrogen) and Illumina's small RNA RT-Primer (5′-CAAGCAGAAGACGGCATACGA-3′) following the manufacturer's instructions. The resulting cDNA was PCR-amplified with Phusion® Hot Start DNA Polymerase (Finnzymes, Espoo, Finland) for 15 cycles using Illumina's small RNA primer set (5′-CAAGCAGAAGACGGCATACGA-3′; 5′-AATGATACGGCGACCACCGA-3′).

The amplification products were purified using a 6% Novex TBE gel (Invitrogen) and the 80–140 nucleotide fractions were excised and eluted in 100 µl of elution buffer (NEB) for 2 h at RT. The resulting gel slurry was passed through a Spin-X filter and precipitated by the addition of 325 µl of 100% ethanol, 10 µl of 3 M sodium acetate, and 1 µl glycogen (20 mg/ml). The samples were centrifuged at 14000 rpm for 20 minutes. After washing with 70% ethanol, the pellet was dried in a SpeedVac (Thermo Fisher Scientific) and dissolved in 10 µl of sequencing grade water. cDNA was PCR-amplified with Phusion® Hot Start DNA Polymerase (Finnzymes, Espoo, Finland) for 15 cycles using Illumina's small RNA primer set (5′-CAAGCAGAAGACGGCATACGA-3′ and 5′- AATGATACGGCGACCACCGACAGGTTCAGAGTTCTACAGTCCGA-3′). Purified PCR products were quantified by Bioanalyzer DNA 1000 chip (Agilent).

### Sequencing of small RNA libraries

Small RNA libraries were diluted in sequencing grade water to obtain 10 nM concentration. A total of eight flow cell lanes were used to sequence the following samples: 1) FCx with Illumina index # 3, 2) FCx with Illumina index # 7, 3) FCx with Illumina index # 11, 4) a pool of 1–3, including 3.3 nM concentration of each sample, 5) HP with Illumina index # 3, 6) HP with Illumina index # 7, 7) HP with Illumina index # 11, and 8) a pool of 5–7, including 3.3 nM concentration of each sample (Figure S1). Sequencing was performed with the Illumina Cluster Station and Genome Analyzer *II* according to manufacturer's protocols using 36 alternating cycles of enzymatic synthesis and optical interrogation. Sequence reads were extracted from the image files using the GAPipeline software v 1.4.0 (Illumina San Diego, CA, USA).

### Small RNA sequence data analysis

Only reads with an index sequence were retained, and in case of the pooled samples sequences were divided into separate files according to the index. Reads were trimmed by removing the index sequence and the 3′ adaptor sequence. Subsequently, sequences including adaptor dimers, mitochondrial or ribosomal sequences were discarded. Additionally, reads that contained homopolymers (i.e., one nucleotide appearing more than 80% of the entire short read) or were shorter than 14 nt were removed. The resulting set of trimmed reads were then mapped against the mouse genome (Mus_musculus.NCBIM37.55) and to known mature miRNAs (miRBase version 11; April 15, 2008; http://www.mirbase.org/) [48], [49], [50], [51]. Version 11 of miRBase was selected because it was used in the probe-design of the Affymetrix microarray. The alignments were performed using Bowtie [52] allowing for two mismatches, because this generated the highest correlation between the miRNA-Seq and Affymetrix microarray data (data not shown). MiRNAs detected with only one count were eliminated from further analyses. Expression analysis of miRNA-Seq data was performed with the R/Bioconductor package *EdgeR* [53], which is designed for use with digital gene expression data. *EdgeR* was chosen because it moderates common dispersion in miRNA-Seq data in a complementary fashion to the *limma* package moderation of probe-wise variation in microarray data. Count numbers of each miRNA were imported to *EdgeR*, log2 transformed, and normalized based on negative binomial distribution to obtain a normalized miRNA expression levels (Table S3). This dataset is available through GEO with an accession number GSE27979. Differential expression of miRNAs between FCx and HP was assessed in *EdgeR* by calculating an exact test p-value analogous to the Fisher's exact test. The correlation analysis of differently indexed libraries was performed with nonparametric Spearman's test, because miRNA-Seq data was not normally distributed.

### Affymetrix miRNA microarray

Oneµg of three FCx and three HP samples was labeled with FlashTag™ Biotin RNA Labeling Kit (Genisphere, Hatfield, PA, USA) for Affymetrix GeneChip® miRNA arrays (Affymetrix, Santa Clara, CA, USA) according to the manufacturer's recommendations. A simple colorimetric Enzyme Linked Oligosorbent Assay (ELOSA) was used to confirm successful biotin labeling. After labeling, the samples were hybridized on Affymetrix GeneChip® miRNA arrays, washed, stained, and scanned according to manufacturer's instructions (Affymetrix, Santa Clara, CA, USA). The data files (.CEL) were imported to R/Bioconductor [54]. The *affy* package [55] was used for preprocessing the raw data with all probes for mouse miRNAs. The RMA method [56] was used to perform background adjustment, quantile normalization and summarization of the log-expression values for each gene on each array (Table S4). This dataset is available through GEO with an accession number GSE27891. We performed a differential expression analysis using methods implemented in the *limma* package, which uses an empirical Bayes method to moderate the standard errors of the estimated log2-fold changes (logFC) [57]. We determined the present/ absent calls with Affymetrix miRNA QC tool, and selected those miRNAs in each brain region with ≥2 replicates detected as present for further analyses.

### Comparison of the expression level of miRNAs across platforms

Comparison of the miRNA expression levels measured by miRNA-Seq and microarray was performed with the *OrderedList* package [58] implemented in R/Bioconductor. For this test, we ranked miRNAs from miRNA-Seq and microarray data separately based on their expression levels in FCx and in HP (normalized count numbers in miRNA-Seq and normalized signal intensity in microarray data). *OrderedList* calculates similarity scores between two ordered lists and determines empirical p-values for the similarity. When computing the similarity score, more weight was given to miRNAs at the ends of the lists. The significance of similarity scores was estimated from random scores with 1000 permutations.

### Visualization of miRNA-Seq reads

Support for viewing miRNA-Seq data was added to the open source software Chipster Viewer. The genomic alignments of the reads were visualized using the Ensembl release 59 annotations and NCBI m37 mouse genome as a reference.

### Target prediction

MiRNAs from both platforms with significantly different expression (p<0.05) between FCx and HP were selected for target prediction. Currently, there exists no single bioinformatic tool with statistically significant accuracy (i.e. low false positive rates) in predicting miRNA binding sites. However, integration of various computational methods is a common approach to improve prediction accuracy and to create an optimal framework for deciphering biological functions of miRNAs [59]. We used the *miRWalk* database (http://www.ma.uni-heidelberg.de/apps/zmf/mirwalk/), which takes into account these issues. *MiRWalk*'*s* major function is to report predicted miRNA-mRNA interactions on the 3′ UTRs of known genes calculated by several established target prediction programs. Of the programs available within the miRWalk database, *TargetScan* [60] and *miRanda* [61] were selected for being well-established algorithms of seed and sequence complementarity with conservation of binding sites across multiple species. Three additional programs were chosen to represent newer but increasingly popular methods for target prediction. *RNA22* [62] first identifies putative miRNA binding sites in the sequence of interest following identification of targeting miRNAs. *RNAhybrid* [63] uses a thermodynamic approach to find energetically favorable hybridization sites between a set of miRNAs and candidate targets. *MiRDB* [64] is a database containing genes predicted to be downregulated by miRNAs with *MiRTarget2*, a prediction program based on support vector machines. The five selected target prediction algorithms represent a unique combination of specificity and sensitivity to potential miRNA-mRNA target gene interactions. Statistically significant miRNA-mRNA relationships were extracted from the *MiRWalk* results using two criteria: an independently calculated Poisson p-value <0.05 for multiple binding sites in a predicted gene, and identification by at least two of the five selected target prediction algorithms.

### Pathway analysis of target genes

Lists of predicted target genes for the differentially expressed miRNA families or clusters were used for input to *Ingenuity Pathway Analysis* (Ingenuity® Systems, http://www.ingenuity.com) with default parameters but a restriction on the results to only the mouse species. This identified known and most relevant biological functions, pathways and annotations in each enriched set of mouse target genes. Further analysis with the tools (as described in the Results) identified biological characteristics such as co-expressional relationships between miRNAs, and assisted in forming statistically supported hypotheses of the true functional roles of miRNAs targeting key genes in a biological pathway.

## Supporting Information

Figure S1 **Sample preparation and sequencing workflow of miRNA-Seq.** Frontal cortex and hippocampi from three adult C57BL/6J mice were dissected and pooled after which total RNA was extracted. Three miRNA libraries were generated from both frontal cortex and hippocampus. Each library was tagged with a different 6-nucleotide index sequence. Eight flow cell lanes were used for sequencing, containing the three frontal cortex and three hippocampus libraries and from both brain regions a pooled sample in which the three indexed libraries were run in 1:3.
(PDF)

Figure S2 **The effect of indexing on the sample similarity.** Count numbers of each miRNA were imported to *EdgeR*, log2 transformed, and normalized based on negative binomial distribution to obtain a relative miRNA expression level. Correlation coefficient was calculated with nonparametric Spearman's test. Index 3, 7, and 11 refer to libraries in which all sequences have this particular index. Single lanes contain averaged information of indexes 3, 7, and 11 (3 technical replicates). Pooled lanes refer to the library in which the same sample with three different indexes was run in a single flow cell lane. Merged lanes contain information from the three individually ran libraries (single lanes), and the pooled library (6 technical replicates). Green boxes indicate FCx libraries and red boxes HP libraries.
(PDF)

Table S1 **MiRNAs differentially expressed between frontal cortex and hippocampus.** Differentially expressed (P<0.05) miRNAs between frontal cortex and hippocampus. FCx  =  frontal cortex, Chr  =  chromosome, Start  =  start position of the sequence in genomic alignment, End  =  end position of the sequence in genomic alignment, AS  =  antisense, S  =  sense, MS  =  miRNA-Seq, MA  =  microarray. MiRNA-Seq count concentration refers to normalized expression levels from edgeR.
(XLSX)

Table S2 **Pathways of predicted target genes for miRNA clusters and families detected as differentially expressed between FCx and HP.** Target genes were predicted using miRWALK for miRNAs belonging to the differentially expressed families/clusters. IPA software was used to identify biological pathways over-represented within the target genes of each family/cluster. Overlap is the number of targets in the dataset over the total number of molecules in the pathway.
(PDF)

Table S3 **MiRNA expression levels detected by miRNA-Seq.** ConcFCx and ConcHP are the normalized expression levels from edgeR for Frontal Cortex (FCx) and Hippocampus (HP). LogFC is the log-transformed fold change of HP over FCx. P-value is calculated by edgeR and is analogous to a p-value from the Fisher's Exact Test. BenjHoch-PVal is the p-value after multiple testing correction using the Benjamini-Hochberg method. Lane values are expression levels normalized to the geometric mean of each library by edgeR.
(XLSX)

Table S4 **Expression levels of miRNAs detected by microarrays.** LogFC refers to the logarithmic fold change calculated as hippocampus over frontal cortex. P-value is given for differential expression. Expression values for each sample represent normalized log-transformed signal intensities.
(XLSX)
