# Supplementary material for: MicroRNA Expression Profiling Reveals MiRNA Families Regulating Specific Biological Pathways in Mouse Frontal Cortex and Hippocampus
Source: PLoS One. 2011 Jun 22;6(6):e21495. doi: 10.1371/journal.pone.0021495 (PMC3120887; doi:10.1371/journal.pone.0021495)
Supplement: Figure S1 — Sample preparation and sequencing workflow of miRNA-Seq. Frontal cortex and hippocampi from three adult C57BL/6J mice were dissected and pooled after which total RNA was extracted. Three miRNA libraries were generated from both frontal cortex and hippocampus. Each library was tagged with a different 6-nucleotide index sequence. Eight flow cell lanes were used for sequencing, containing the three frontal cortex and three hippocampus libraries and from both brain regions a pooled sample in which the three indexed libraries were run in 1:3. (PDF) [file pone.0021495.s001.pdf]

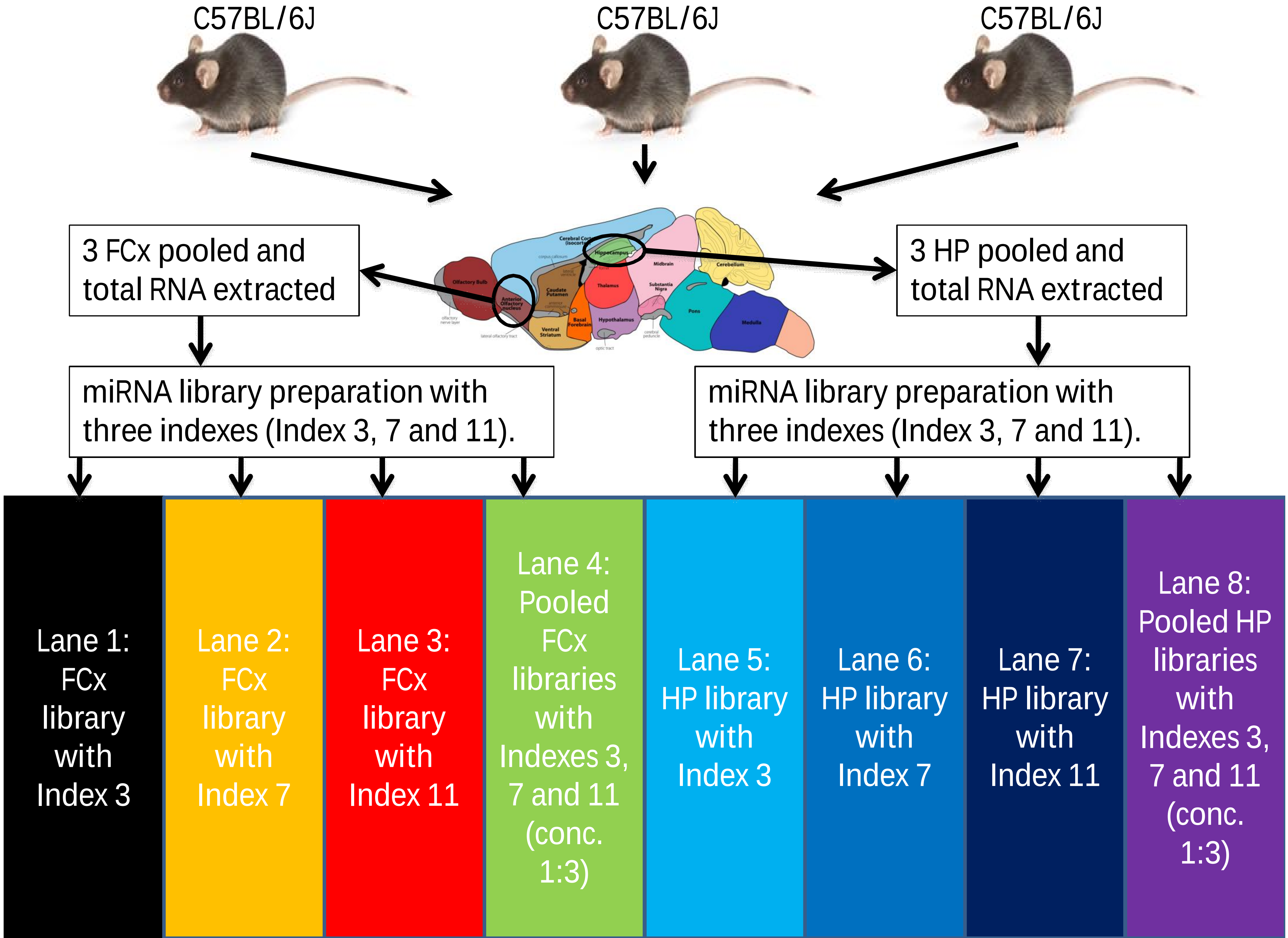
C57BL/6J
C57BL/6J
C57BL/6J
3 FCx pooled and total RNA extracted
3 HP pooled and total RNA extracted
Olfactory Bulb
Anterior Olfactory nucleus
Hippocampus
Midbrain
Cerebellum
Thalamus
Caudate Putamen
Substantia Nigra
Hypothalamus
Basal Forebrain
Ventral Striatum
Pons
Medulla
miRNA library preparation with three indexes (Index 3, 7 and 11).
miRNA library preparation with three indexes (Index 3, 7 and 11).
Lane 1: FCx library with Index 3
Lane 2: FCx library with Index 7
Lane 3: FCx library with Index 11
Lane 4: Pooled FCx libraries with Indexes 3, 7 and 11 (conc. 1:3)
Lane 5: HP library with Index 3
Lane 6: HP library with Index 7
Lane 7: HP library with Index 11
Lane 8: Pooled HP libraries with Indexes 3, 7 and 11 (conc. 1:3)
