# Supplementary figures and images for: MicroRNA Expression Profiling Reveals MiRNA Families Regulating Specific Biological Pathways in Mouse Frontal Cortex and Hippocampus
Source: PLoS One. 2011 Jun 22;6(6):e21495. doi: 10.1371/journal.pone.0021495 (PMC3120887; doi:10.1371/journal.pone.0021495)

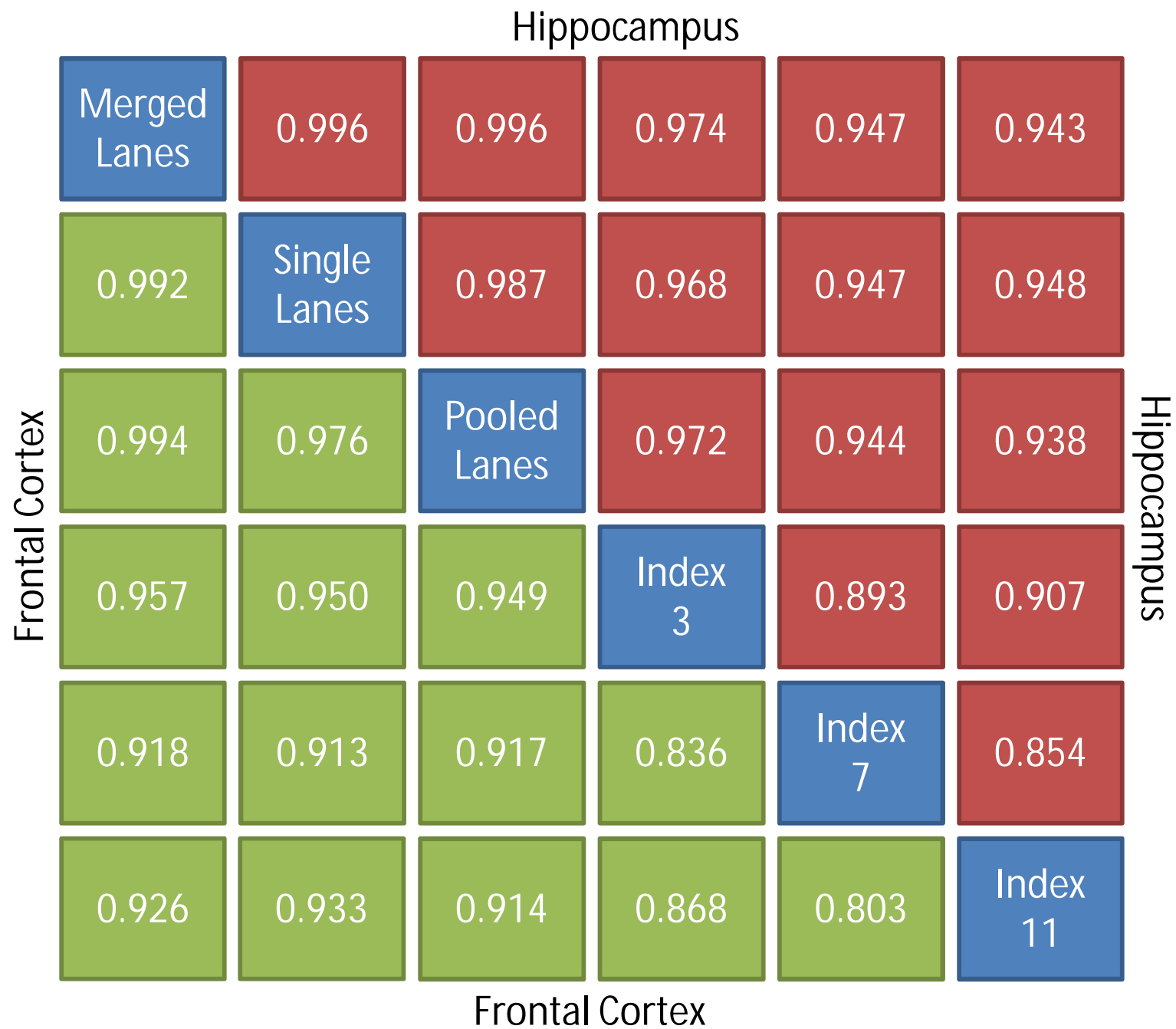

Supplement: Figure S2 — The effect of indexing on the sample similarity. Count numbers of each miRNA were imported to EdgeR, log2 transformed, and normalized based on negative binomial distribution to obtain a relative miRNA expression level. Correlation coefficient was calculated with nonparametric Spearman's test. Index 3, 7, and 11 refer to libraries in which all sequences have this particular index. Single lanes contain averaged information of indexes 3, 7, and 11 (3 technical replicates). Pooled lanes refer to the library in which the same sample with three different indexes was run in a single flow cell lane. Merged lanes contain information from the three individually ran libraries (single lanes), and the pooled library (6 technical replicates). Green boxes indicate FCx libraries and red boxes HP libraries. (PDF) [file pone.0021495.s002.pdf]
