# Supplementary material for: MicroRNA Expression Profiling Reveals MiRNA Families Regulating Specific Biological Pathways in Mouse Frontal Cortex and Hippocampus
Source: PLoS One. 2011 Jun 22;6(6):e21495. doi: 10.1371/journal.pone.0021495 (PMC3120887; doi:10.1371/journal.pone.0021495)
Supplement: Table S2 — Pathways of predicted target genes for miRNA clusters and families detected as differentially expressed between FCx and HP. Target genes were predicted using miRWALK for miRNAs belonging to the differentially expressed families/clusters. IPA software was used to identify biological pathways over-represented within the target genes of each family/cluster. Overlap is the number of targets in the dataset over the total number of molecules in the pathway. (PDF) [file pone.0021495.s004.pdf]

Supplemental Table S2. Pathways of predicted target genes for miRNA clusters and families detected as differentially expressed between FCx and HP. Target genes were predicted using miRWALK for miRNAs belonging to the differentially expressed families/clusters. IPA software was used to identify biological pathways over-represented within the target genes of each family/cluster. Overlap is the number of targets in the dataset over the total number of molecules in the pathway.

Mir-8 Family	P-Value	Target Genes	Overlap
Ephrin Receptor Signaling	3,02E-03	*Rap1b, Epha7, Gnai3, Efnb2, Wipf1, Angpt1, Cfl2, Cxcl12, Mras, Map4k4, Epha2, Efna1*	12/186 (6%)
Axonal Guidance Signaling	6,76E-03	*Epha7, Rap1b, Prkacb, Sema6a, Slit2, Efna1, Efnb2, Sema3a, Gnai3, Wipf1, Nfat5, Ntrk2, Adam12, Gli3, Cxcl12, Cfl2, Mknk1, Mras, Epha2*	19/381 (5%)
Ceramide Signaling	7,24E-03	*Map2k4, Jun, Ppp2r5c, Map3k1, Mras, Ppp2r2c, Nsmaf*	7/81 (9%)
Cdk5 Signaling	1,00E-02	*Prkacb, Fosb, Ntrk2, Ppp2r5c, Mras, Ppp2r2c, Cdk5r1*	7/86 (8%)
Toll-Like Receptor Signaling	1,10E-02	*Map2k4, Tlr4, Jun, Map3k1, Map4k4*	5/48 (10%)
Erk/Mapk Signaling	1,35E-02	*Rap1b, Prkacb, Mycn, Ywhag, Ppp2r5c, Dusp1, Mknk1, Mras, Ywhaz, Ppp2r2c, Elf5*	11/191 (6%)
Cell Cycle Regulation By Btg Family Proteins	1,48E-02	*Ccne2, Ppp2r5c, Ppp2r2c, E2f3*	4/34 (12%)
Ascorbate And Aldarate Metabolism	1,78E-02	*Aldh1b1, Aldh1a1, Aldh1a7*	3/20 (15%)
April Mediated Signaling	2,14E-02	*Map2k4, Nfat5, Jun, Map3k1*	4/40 (10%)
Lysine Degradation	2,19E-02	*Aldh1b1, Bbox1, Aldh1a1, Aldh1a7, Suv39h2*	5/57 (9%)
P70s6k Signaling	2,19E-02	*Gnai3, Ywhag, F2r, Ppp2r5c, Mras, Ywhaz, Ppp2r2c, Egfr*	8/125 (6%)
B Cell Activating Factor Signaling	2,57E-02	*Map2k4, Nfat5, Jun, Map3k1*	4/42 (10%)
Rac Signaling	2,63E-02	*Map2k4, Tiam1, Jun, Cfl2, Map3k1, Mras, Cdk5r1*	7/113 (6%)
Glycosphingolipid Biosynthesis - Neolactoseries	2,88E-02	*Gcnt2, St3gal6, B3gnt1*	3/24 (13%)
Production Of Nitric Oxide And Reactive Oxygen Species In Macrophages	2,88E-02	*Map2k4, Rap1b, Tlr4, Jun, Ppp2r5c, Rnd3, Map3k1, Ifngr2, Ppp2r2c*	9/158 (6%)
Erk5 Signaling	3,02E-02	*Ywhag, Mras, Ywhaz, Wnk1, Egfr*	5/64 (8%)
Chondroitin Sulfate Biosynthesis	3,72E-02	*Chsy1, Hs3st1, Chst10, Ndst1*	4/45 (9%)
Agrin Interactions At Neuromuscular Junction	3,80E-02	*Map2k4, Jun, Mras, Gabpa, Egfr*	5/66 (8%)
Tnfr1 Signaling	3,98E-02	*Map2k4, Jun, Casp2, Map3k1*	4/48 (8%)
Egf Signaling	3,98E-02	*Map2k4, Jun, Map3k1, Egfr*	4/47 (9%)
Il-1 Signaling	4,17E-02	*Map2k4, Prkacb, Gnai3, Jun, Map3k1, Mras*	6/95 (6%)
Keratan Sulfate Biosynthesis	4,27E-02	*Hs3st1, Chst10, Ndst1, B3gnt1*	4/47 (9%)
Gaba Receptor Signaling	4,27E-02	*Slc6a11, Slc6a1, Gphn, Ap2a2*	4/47 (9%)
Tnfr2 Signaling	4,27E-02	*Map2k4, Jun, Map3k1*	3/30 (10%)
4-1bb Signaling In T Lymphocytes	4,68E-02	*Map2k4, Jun, Map3k1*	3/31 (10%)
Hmgb1 Signaling	4,68E-02	*Map2k4, Tlr4, Jun, Rnd3, Mras, Ifngr2*	6/94 (6%)
**Mir-182, Mir-183 And Mir-96 Cluster**	**P-Value**	**Target Genes**	**Overlap**
Alpha-Adrenergic Signaling	2,95E-05	*Prkacb, Adra2a, Gnai3, Mras, Adcy6, Gnaq, Prkch, Gng5, Map2k1, Gng4, Prkar1a, Gys2*	12/90 (13%)
P2y Purigenic Receptor Signaling Pathway	1,17E-04	*Prkacb, Rac1, Adcy6, Gnaq, Gng5, Pik3r3, Gnai3, Creb1, Mras, Prkch, Map2k1, Gng4, Prkar1a*	13/117 (11%)
Ephrin Receptor Signaling	1,55E-04	*Gnaq, Rac1, Limk2, Epha4, Epha3, Gng5, Efnb2, Gnai3, Ephb1, Wipf1, Wasl (Includes Eg:8976), Sdc2, Creb1, Mras, Gng4, Map2k1*	16/185 (9%)
Igf-1 Signaling	1,95E-04	*Prkacb, Pik3r3, Ywhag, Foxo1, Irs1, Foxo3, Mras, Rac1, Prkch, Map2k1, Prkar1a*	11/93 (12%)
Role Of Nfat In Cardiac Hypertrophy	3,24E-04	*Prkacb, Gnaq, Rac1, Adcy6, Hand1, Hdac9 (Includes Eg:9734), Gng5, Pik3r3, Gnai3, Ppp3r1, Mras, Prkch, Mef2c, Gng4, Map2k1, Prkar1a*	16/188 (9%)
Cardiac Hypertrophy Signaling	3,55E-04	*Prkacb, Gnaq, Adcy6, Hand1, Gng5, Pik3r3, Adra2a, Gnai3, Ppp3r1, Irs1, Creb1, Mras, Adra2c, Mef2c, Gng4, Map2k1, Tab1, Prkar1a*	18/221 (8%)
Breast Cancer Regulation By Stathmin1	3,89E-04	*Prkacb, Ppp2r5c, Gnaq, Rac1, Adcy6, Limk2, Gng5, Pik3r3, Ppp2cb, Gnai3, Mras, Prkch, Arhgef3, Gng4, Map2k1, Prkar1a*	16/184 (9%)
Phospholipase C Signaling	3,89E-04	*Napepld, Rala, Gnaq, Rac1, Adcy6, Hdac9 (Includes Eg:9734), Gng5, Cd3d, Pld1, Nfat5, Ppp3r1, Creb1, Mras, Mef2c, Prkch, Arhgef3, Gng4, Map2k1*	18/232 (8%)
Axonal Guidance Signaling	4,07E-04	*Prkacb, Gli2, Epha4, Limk2, Efnb2, Ephb1, Wasl (Includes Eg:8976), Nfat5, Sdc2, Ppp3r1, Dcc, Mras, Gng4, Map2k1, Rac1, Pfn2, Gnaq, Epha3, Gng5, Pik3r3, Gnai3, Dock1, Wipf1, Prkch, Prkar1a*	25/378 (7%)
Pi3k/Akt Signaling	4,47E-04	*Pik3r3, Ppp2cb, Ywhag, Foxo1, Ppp2r5c, Foxo3, Mras, Rac1, Lims1, Inppl1, Map2k1, Gys2*	12/126 (10%)
P70s6k Signaling	6,61E-04	*Pik3r3, Ppp2cb, Gnai3, Ywhag, Ppp2r5c, Irs1, Mras, Rac1, Gnaq, Prkch, Map2k1, Pld1*	12/123 (10%)
G Beta Gamma Signaling	6,76E-04	*Prkacb, Gnai3, Mras, Rac1, Gnaq, Hbegf, Prkch, Gng5, Gng4, Prkar1a*	10/97 (10%)

Relaxin Signaling	8,71E-04	*Prkacb, Pik3r3, Gnai3, Creb1, Mras, Rac1, Adcy6, Gnaq, Gng5, Map2k1, Gng4, Prkar1a*	12/130 (9%)
Insulin Receptor Signaling	1,10E-03	*Prkacb, Pik3r3, Foxo1, Irs1, Foxo3, Mras, Rac1, Inppl1, Prkch, Map2k1, Prkar1a, Gys2*	12/128 (9%)
Creb Signaling In Neurons	1,26E-03	*Prkacb, Gria1, Rac1, Adcy6, Gnaq, Gng5, Pik3r3, Gnai3, Creb1, Mras, Prkch, Map2k1, Gng4, Prkar1a*	14/177 (8%)
Melanocyte Development And Pigmentation Signaling	1,59E-03	*Prkacb, Pik3r3, Rps6ka6, Mitf, Creb1, Mras, Adcy6, Map2k1, Prkar1a*	9/83 (11%)
Il-4 Signaling	1,66E-03	*Pik3r3, Stat6, Nfat5, Il13ra1, Irs1, Mras, Rac1, Inppl1*	8/68 (12%)
Protein Kinase A Signaling	1,74E-03	*Prkacb, Ywhag, Gnaq, Adcy6, Akap6, Gng5, Akap7, Gys2, Gnai3, Nfat5, Add3, Ppp3r1, Creb1, Dcc, Prkch, Kdelr1, Gng4, Map2k1, Tcf7l2 (Includes Eg:6934), Prkar1a*	20/294 (7%)
Camp-Mediated Signaling	1,95E-03	*Prkacb, Rgs2, Htr4, Adcy6, Akap6, Akap7, Adra2a, Ppp3r1, Creb1, Adra2c, Htr1a, Map2k1, Prkar1a*	13/155 (8%)
Factors Promoting Cardiogenesis In Vertebrates	2,24E-03	*Tgfbr3, Lrp6, Acvr1, Tdgf1, Mef2c, Prkch, Smad1, Acvr1c, Tcf7l2 (Includes Eg:6934)*	9/88 (10%)
Il-3 Signaling	2,40E-03	*Pik3r3, Stat6, Foxo1, Ppp3r1, Mras, Rac1, Prkch, Map2k1*	8/72 (11%)
Ppar-Alpha/Rxr-Alpha Activation	2,40E-03	*Prkacb, Med1, Tgfbr3, Acvr1, Adcy6, Gnaq, Gk, Irs1, Mras, Clock, Map2k1, Acvr1c, Prkar1a*	13/164 (8%)
Fc-Gamma Receptor-Mediated Phagocytosis In Macrophages And Monocytes	2,57E-03	*Pik3r3, Dock1, Napepld, Ezr, Rac1, Vamp3, Tln1, Prkch, Pld1*	9/90 (10%)
Fmlp Signaling In Neutrophils	2,63E-03	*Pik3r3, Gnai3, Nfat5, Ppp3r1, Mras, Rac1, Prkch, Gng5, Map2k1, Gng4*	10/111 (9%)
Il-1 Signaling	2,82E-03	*Prkacb, Gnai3, Mras, Adcy6, Gnaq, Gng5, Gng4, Tab1, Prkar1a*	9/94 (10%)
G-Protein Coupled Receptor Signaling	3,31E-03	*Prkacb, Rgs2, Ednrb, Htr4, Gnaq, Rac1, Adcy6, Adra2a, Pik3r3, Creb1, Mras, Adra2c, Htr1a, Map2k1, Prkar1a*	15/206 (7%)
Corticotropin Releasing Hormone Signaling	3,47E-03	*Prkacb, Gnai3, Gli2, Creb1, Adcy6, Gnaq, Mef2c, Prkch, Map2k1, Prkar1a*	10/116 (9%)
Role Of Nfat In Regulation Of The Immune Response	3,55E-03	*Csnk1g3, Rac1, Gnaq, Gng5, Cd3d, Pik3r3, Gnai3, Nfat5, Ppp3r1, Mras, Mef2c, Map2k1, Gng4*	13/177 (7%)
Molecular Mechanisms Of Cancer	3,63E-03	*Prkacb, Rala, Lrp6, Gnaq, Adcy6, Rac1, Psen2, Pik3r3, Gnai3, Nf1, Foxo1, Irs1, Mras, Prkch, Arhgef3, Pmaip1, Map2k1, Smad1, Tab1, Prkar1a, Atm*	21/347 (6%)
Integrin Signaling	3,80E-03	*Capn6, Parva, Rala, Rac1, Tln1, Lims1, Tnk2, Pik3r3, Dock1, Wipf1, Wasl (Includes Eg:8976), Arf4, Mras, Map2k1*	14/195 (7%)
Ampk Signaling	4,07E-03	*Prkacb, Adra2a, Pik3r3, Ppp2cb, Ppp2r5c, Irs1, Mras, Rac1, Adra2c, Prkar1a, Gys2*	11/136 (8%)
Erk5 Signaling	4,37E-03	*Rps6ka6, Ywhag, Creb1, Foxo3, Mras, Gnaq, Mef2c*	7/64 (11%)
Cardiac Beta-Adrenergic Signaling	6,03E-03	*Prkacb, Ppp2cb, Ppp2r5c, Mras, Adcy6, Akap6, Gng5, Gng4, Akap7, Prkar1a*	10/121 (8%)
Gnrh Signaling	6,46E-03	*Prkacb, Gnai3, Creb1, Mras, Rac1, Adcy6, Gnaq, Prkch, Map2k1, Prkar1a*	10/125 (8%)
Synaptic Long Term Potentiation	6,92E-03	*Prkacb, Gria1, Ppp3r1, Creb1, Mras, Gnaq, Prkch, Map2k1, Prkar1a*	9/102 (9%)
Cdk5 Signaling	7,24E-03	*Prkacb, Lamc1, Ppp2cb, Ppp2r5c, Mras, Adcy6, Map2k1, Prkar1a*	8/86 (9%)
Pak Signaling	7,24E-03	*Pik3r3, Wasl (Includes Eg:8976), Mras, Rac1, Dscam, Limk2, Epha3, Map2k1*	8/97 (8%)
Renin-Angiotensin Signaling	7,76E-03	*Prkacb, Pik3r3, Mras, Rac1, Adcy6, Gnaq, Prkch, Map2k1, Prkar1a*	9/105 (9%)
Wnt/Beta-Catenin Signaling	8,13E-03	*Ppp2cb, Ppp2r5c, Csnk1g3, Tgfbr3, Lrp6, Acvr1, Rac1, Gnaq, Rarg, Acvr1c, Tcf7l2 (Includes Eg:6934), Tab1*	12/163 (7%)
Amyloid Processing	8,13E-03	*Prkacb, Capn6, Rac1, Psen2, Bace2, Prkar1a*	6/54 (11%)
Regulation Of Eif4 And P70s6k Signaling	8,32E-03	*Pik3r3, Ppp2cb, Ppp2r5c, Eif4ebp2, Irs1, Mras, Rac1, Paip2, Map2k1*	9/118 (8%)
Pten Signaling	8,32E-03	*Pik3r3, Magi1, Foxo1, Foxo3, Mras, Rac1, Inppl1, Map2k1*	8/96 (8%)
Il-8 Signaling	8,91E-03	*Pik3r3, Gnai3, Napepld, Mras, Rac1, Hbegf, Limk2, Prkch, Gng5, Map2k1, Gng4, Pld1*	12/169 (7%)
Bmp Signaling Pathway	9,33E-03	*Prkacb, Creb1, Mras, Smad1, Map2k1, Tab1, Prkar1a*	7/73 (10%)
Dopamine Receptor Signaling	1,00E-02	*Prkacb, Ppp2cb, Ppp2r5c, Adcy6, Qdpr, Maoa, Prkar1a*	7/72 (10%)
Ccr3 Signaling In Eosinophils	1,05E-02	*Pik3r3, Gnai3, Mras, Rac1, Limk2, Prkch, Gng5, Map2k1, Gng4*	9/113 (8%)
Androgen Signaling	1,05E-02	*Prkacb, Gnai3, Myst2, Mras, Gnaq, Prkch, Gng5, Gng4, Prkar1a*	9/120 (8%)
Cxcr4 Signaling	1,12E-02	*Pik3r3, Dock1, Gnai3, Mras, Rac1, Adcy6, Gnaq, Prkch, Gng5, Map2k1, Gng4*	11/153 (7%)
Flt3 Signaling In Hematopoietic Progenitor Cells	1,15E-02	*Pik3r3, Stat6, Rps6ka6, Creb1, Mras, Rac1, Map2k1*	7/75 (9%)
Leptin Signaling In Obesity	1,15E-02	*Prkacb, Pik3r3, Foxo1, Rac1, Adcy6, Map2k1, Prkar1a*	7/75 (9%)
Mtor Signaling	1,48E-02	*Pik3r3, Ppp2cb, Rps6ka6, Napepld, Ppp2r5c, Irs1, Mras, Rac1, Prkch, Pld1*	10/141 (7%)

Melanoma Signaling	1,51E-02	Pik3r3, Mitf, Mras, Rac1, Map2k1	5/45 (11%)
Serotonin Receptor Signaling	1,66E-02	Htr4, Htr1a, Qdpr, Maoa	4/30 (13%)
O-Glycan Biosynthesis	1,86E-02	Galnt7, Galntl1, Galnt2, Fut4	4/31 (13%)
Ccr5 Signaling In Macrophages	2,09E-02	Gnai3, Mras, Prkch, Gng5, Cd3d, Gng4	6/77 (8%)
G Protein Signaling Mediated By Tubby	2,09E-02	Mras, Gnaq, Gng5, Gng4	4/35 (11%)
Thrombin Signaling	2,14E-02	Pik3r3, Gnai3, Creb1, Mras, Rac1, Adcy6, Gnaq, Prkch, Arhgef3, Gng5, Map2k1, Gng4	12/189 (6%)
Rhoa Signaling	2,14E-02	Ktn1, Ezr, Rdx, Pfn2, Arhgap12, Rapgef6, Limk2, Pld1	8/103 (8%)
Melatonin Signaling	2,40E-02	Prkacb, Gnai3, Gnaq, Prkch, Map2k1, Prkar1a	6/67 (9%)
Non-Small Cell Lung Cancer Signaling	2,40E-02	Pik3r3, Rassf1, Foxo3, Mras, Rac1, Map2k1	6/73 (8%)
Fak Signaling	2,57E-02	Pik3r3, Dock1, Capn6, Mras, Rac1, Tln1, Map2k1	7/96 (7%)
Neuregulin Signaling	2,75E-02	Pik3r3, Erbb2ip (Includes Eg:55914), Mras, Rac1, Hbegf, Prkch, Map2k1	7/96 (7%)
Lps-Stimulated Mapk Signaling	2,88E-02	Pik3r3, Creb1, Mras, Rac1, Prkch, Map2k1	6/73 (8%)
Sapk/Jnk Signaling	3,02E-02	Pik3r3, Irs1, Dusp10, Mras, Rac1, Gng5, Tab1	7/95 (7%)
Pi3k Signaling In B Lymphocytes	3,09E-02	Nfat5, Irs1, Ppp3r1, Creb1, Foxo3, Mras, Rac1, Prkch, Map2k1	9/134 (7%)
Erk/Mapk Signaling	3,16E-02	Prkacb, Pik3r3, Ppp2cb, Ywhag, Ppp2r5c, Creb1, Mras, Rac1, Tln1, Map2k1, Prkar1a	11/184 (6%)
Natural Killer Cell Signaling	3,24E-02	Pik3r3, Sh2d1a, Mras, Rac1, Inppl1, Prkch, Map2k1	7/92 (8%)
Clathrin-Mediated Endocytosis Signaling	3,24E-02	Pik3r3, Wasl (Includes Eg:8976), Ldlr, Fgf9, Ppp3r1, Amph, Rac1, Sh3glb1, Sh3kbp1, Cttn	10/159 (6%)
Antiproliferative Role Of Somatostatin Receptor 2	3,24E-02	Pik3r3, Mras, Adcy6, Gng5, Map2k1, Gng4	6/74 (8%)
Endometrial Cancer Signaling	3,31E-02	Pik3r3, Foxo3, Mras, Rac1, Map2k1	5/56 (9%)
Neuroprotective Role Of Thop1 In Alzheimer's Disease	3,72E-02	Prkacb, Mme, Creb1, Prkar1a	4/39 (10%)
Leukocyte Extravasation Signaling	3,72E-02	Pik3r3, Gnai3, Wipf1, Wasl (Includes Eg:8976), Cldn1, Ezr, Rac1, Rdx, Arhgap12, Prkch, Cttn	11/180 (6%)
Synaptic Long Term Depression	3,80E-02	Ppp2cb, Gnai3, Ppp2r5c, Gria1, Mras, Adcy6, Gnaq, Prkch, Map2k1	9/142 (6%)
Actin Cytoskeleton Signaling	4,07E-02	Pik3r3, Dock1, Wasl (Includes Eg:8976), Ezr, Fgf9, Mras, Rac1, Rdx, Pfn2, Limk2, Wasf2, Map2k1	12/218 (6%)
T Cell Receptor Signaling	4,27E-02	Pik3r3, Nfat5, Ppp3r1, Mras, Rac1, Map2k1, Cd3d	7/100 (7%)
Tgf-Beta Signaling	4,27E-02	Acvr1, Mras, Smad1, Map2k1, Acvr1c, Tab1	6/79 (8%)
Docosahexaenoic Acid (Dha) Signaling	4,37E-02	Pik3r3, Foxo1, Rac1, Pnpla2	4/41 (10%)
Rar Activation	4,47E-02	Prkacb, Pik3r3, Med1, Rac1, Adcy6, Prkch, Smad1, Map2k1, Rarg, Prkar1a	10/166 (6%)
Acute Myeloid Leukemia Signaling	4,57E-02	Pik3r3, Cebpa, Mras, Rac1, Map2k1, Tcf7l2 (Includes Eg:6934)	6/79 (8%)
Ceramide Signaling	4,79E-02	Pik3r3, Ppp2cb, Ppp2r5c, Mras, Rac1, Map2k1	6/80 (8%)
Il-12 Signaling And Production In Macrophages	4,90E-02	Pik3r3, Stat6, Il12a, Maf, Rac1, Prkch, Map2k1	7/104 (7%)
**Mir-132 Family**	**P-Value**	**Target Genes**	**Overlap**
Regulation Of Eif4 And P70s6k Signaling	7,76E-04	Rps6kb1, Eif2s2, Rras2, Ppp2r4, Paip2, Pik3r4	6/118 (5%)
Hereditary Breast Cancer Signaling	1,32E-03	Rb1, Hdac3, Rras2, Rfc1, Pik3r4, Ep300	6/119 (5%)
Chronic Myeloid Leukemia Signaling	3,09E-03	Rb1, Hdac3, Rras2, E2f5, Pik3r4	5/101 (5%)
Cell Cycle Regulation By Btg Family Proteins	5,13E-03	Rb1, Ppp2r4, E2f5	3/34 (9%)
Ceramide Signaling	9,12E-03	Rras2, Ppp2r4, Pik3r4, Tnfrsf11b	4/80 (5%)
Melanocyte Development And Pigmentation Signaling	1,05E-02	Rps6kb1, Rras2, Pik3r4, Ep300	4/83 (5%)
Melanoma Signaling	1,07E-02	Rb1, Rras2, Pik3r4	3/45 (7%)
Vegf Signaling	1,17E-02	Eif2s2, Rras2, Actb, Pik3r4	4/89 (4%)
P53 Signaling	1,32E-02	Rb1, Rrm2b, Pik3r4, Ep300	4/89 (4%)
Fc-Gamma Receptor-Mediated Phagocytosis In Macrophages And Monocytes	1,32E-02	Rps6kb1, Actr2, Actb, Rab11a	4/90 (4%)
Cntf Signaling	1,51E-02	Rps6kb1, Rras2, Pik3r4	3/52 (6%)
Glioblastoma Multiforme Signaling	1,78E-02	Rps6kb1, Rb1, Rras2, E2f5, Pik3r4	5/151 (3%)
Glioma Signaling	1,82E-02	Rb1, Rras2, E2f5, Pik3r4	4/105 (4%)
Cell Cycle: G1/S Checkpoint Regulation	1,86E-02	Rb1, Hdac3, E2f5	3/58 (5%)
Role Of Brca1 In Dna Damage Response	1,95E-02	Rb1, E2f5, Rfc1	3/55 (5%)
Tight Junction Signaling	2,09E-02	Cldn1, Ppp2r4, Actb, Tnfrsf11b, Ocln	5/153 (3%)
Pancreatic Adenocarcinoma Signaling	2,24E-02	Rb1, E2f5, Hbegf, Pik3r4	4/108 (4%)
Rac Signaling	2,24E-02	Rps6kb1, Actr2, Rras2, Pik3r4	4/113 (4%)
Propanoate Metabolism	3,02E-02	Acat2, Acss2, Acadm	3/65 (5%)
Nrf2-Mediated Oxidative Stress Response	3,02E-02	Sod2, Rras2, Actb, Pik3r4, Ep300	5/176 (3%)
Non-Small Cell Lung Cancer Signaling	3,24E-02	Rb1, Rras2, Pik3r4	3/73 (4%)
Il-4 Signaling	3,24E-02	Rps6kb1, Rras2, Pik3r4	3/68 (4%)
Erythropoietin Signaling	3,39E-02	Rps6kb1, Rras2, Pik3r4	3/72 (4%)
Renal Cell Carcinoma Signaling	3,47E-02	Rras2, Pik3r4, Ep300	3/70 (4%)
Erk/Mapk Signaling	3,47E-02	Dusp9, Rras2, Ppp2r4, Hspb7, Pik3r4	5/184 (3%)

Prolactin Signaling	3,63E-02	*Rras2, Pik3r4, Ep300*	3/73 (4%)
P70s6k Signaling	3,63E-02	*Rps6kb1, Rras2, Ppp2r4, Pik3r4*	4/123 (3%)
Methionine Metabolism	3,89E-02	*Sms, Mat2b*	2/30 (7%)
Flt3 Signaling In Hematopoietic Progenitor Cells	4,17E-02	*Rps6kb1, Rras2, Pik3r4*	3/75 (4%)
Ovarian Cancer Signaling	4,47E-02	*Rps6kb1, Rb1, Rras2, Pik3r4*	4/133 (3%)
Integrin Signaling	4,47E-02	*Rap2b, Actr2, Rras2, Actb, Pik3r4*	5/195 (3%)
Role Of Chk Proteins In Cell Cycle Checkpoint Control	4,57E-02	*E2f5, Rfc1*	2/33 (6%)
Pyruvate Metabolism	4,57E-02	*Pdha1 (Includes Eg:5160), Acat2, Acss2*	3/77 (4%)
Ampk Signaling	4,68E-02	*Rps6kb1, Ppp2r4, Pik3r4, Pfkfb2*	4/136 (3%)
Acute Myeloid Leukemia Signaling	4,79E-02	*Rps6kb1, Rras2, Pik3r4*	3/79 (4%)
Selenoamino Acid Metabolism	4,79E-02	*Acss2, Mat2b*	2/34 (6%)
Regulation Of Actin-Based Motility By Rho	4,90E-02	*Actr2, Actb, Pfn2*	3/80 (4%)

Mir-34 Family	P-Value	Target Genes	Overlap
Cardiac Beta-Adrenergic Signaling	6,31E-03	*Ppp1cc, Prkar2b, Adrbk1, Ppp2r4, Mras, Akap6, Pkia, Ppp2r5a*	8/121 (7%)
Ampk Signaling	1,10E-02	*Prkar2b, Acacb, Pfkfb4, Ppp2r4, Ppm1b, Mras, Lipe, Ppp2r5a*	8/136 (6%)
Notch Signaling	1,12E-02	*Dll1, Jag1, Notch1, Hey1*	4/40 (10%)
Myc Mediated Apoptosis Signaling	1,35E-02	*Ywhag, Mras, Mapk9, Bid, Bax*	5/62 (8%)
Mtor Signaling	1,35E-02	*Rhoq, Ppp2r4, Mras, Fkbp1a, Rps6ka4, Rps6ka5, Eif4e, Ppp2r5a*	8/141 (6%)
Erk5 Signaling	1,45E-02	*Ywhag, Mras, Rps6ka4, Rps6ka5, Map3k3*	5/64 (8%)
Tumoricidal Function Of Hepatic Natural Killer Cells	1,74E-02	*Prf1, Bid, Bax*	3/24 (13%)
Erk/Mapk Signaling	2,04E-02	*Ppp1cc, Ywhag, Prkar2b, Ppp2r4, Mras, Rps6ka4, Rps6ka5, Eif4e, Ppp2r5a*	9/184 (5%)
Molecular Mechanisms Of Cancer	2,29E-02	*Mapk9, Cdkn2c, Bax, Prkar2b, Rhoq, Nf1, Sufu, Rasgrp1, Mras, Bid, Notch1, E2f2, Rap2a, Wnt5a*	14/347 (4%)
Dopamine Receptor Signaling	2,57E-02	*Ppp1cc, Prkar2b, Ppp2r4, Ppp2r5a, Caly*	5/72 (7%)
Sonic Hedgehog Signaling	2,63E-02	*Prkar2b, Adrbk1, Sufu*	3/29 (10%)
Semaphorin Signaling In Neurons	3,02E-02	*Met, Sema3a, Rhoq, Arhgap1*	4/51 (8%)
Wnt/Beta-Catenin Signaling	3,55E-02	*Cdh3, Ppp2r4, Sox12, Sfrp1, Pin1, Rarg, Ppp2r5a, Wnt5a*	8/163 (5%)
Cell Cycle Regulation By Btg Family Proteins	4,37E-02	*Ppp2r4, E2f2, Ppp2r5a*	3/34 (9%)
Cdk5 Signaling	4,79E-02	*Ppp1cc, Prkar2b, Ppp2r4, Mras, Ppp2r5a*	5/86 (6%)
Actin Cytoskeleton Signaling	4,90E-02	*Ppp1cc, Pip5k1a, Vav3, Fgf9, Mras, Rdx, Myh9, Wasf1, Pdgfc*	9/218 (4%)
